# A survey of caregiver acculturation and acceptance of silver diamine fluoride treatment for childhood caries

**DOI:** 10.1186/s12903-019-0915-1

**Published:** 2019-10-24

**Authors:** Anjali Kumar, Dana Cernigliaro, Mary E. Northridge, Yinxiang Wu, Andrea B. Troxel, Joana Cunha-Cruz, Jay Balzer, David M. Okuji

**Affiliations:** 1 NYU Langone Dental Medicine, 5800 Third Avenue, Brooklyn, NY 11220 USA; 2 Division of Biostatistics, Department of Population Health, 650 First Avenue, New York, NY 10016 USA; 3 University of Washington, Box 357475, Seattle, WA 98195 USA

**Keywords:** Health equity, Oral health, Pediatric dentists, Parental consent, Social acceptance, Community health centers

## Abstract

**Background:**

Interest in aqueous silver diamine fluoride (SDF) has been growing as a treatment for caries arrest. A cross-sectional study was conducted to identify factors associated with caregiver acceptance of SDF treatment for children presenting with caries at 8 Federally Qualified Health Centers. The study purpose was to examine associations between caregiver acceptance of SDF treatment for children with caries and (1) sociodemographic and acculturation characteristics of caregivers and (2) clinical assessments of the children by dentists.

**Methods:**

A caregiver survey collected information on: sociodemographic characteristics; acculturation characteristics, measured using the validated Short Acculturation Scale for Hispanics (SASH); perceived benefits and barriers of SDF treatment, including caregiver comfort; and perceived health-related knowledge. Chart reviews were conducted to assess: the medical / dental insurance of pediatric patients; cumulative caries experience, measured using decayed, missing, filled teeth total scores (dmft / DMFT); whether operating room treatment was needed; and a record of caregiver acceptance of SDF treatment (the outcome measure). Standard logistic regression models were developed for caregiver acceptance of SDF treatment for their children as the binary outcome of interest (yes / no) to calculate unadjusted odds ratios (OR) and adjusted ORs for covariates of interest.

**Results:**

Overall, 434 of 546 caregivers (79.5%) accepted SDF treatment for their children. A U-shaped relationship between caregiver odds of accepting SDF treatment and age group of pediatric patients was present, where caregivers were most likely to accept SDF treatment for their children who were either < 6 years or 9–14 years, and least likely to accept SDF treatment for children 6 to < 9 years. The relationship between acculturation and caregiver acceptance of SDF treatment depended upon whether or not caregivers were born in the United States: greater acculturation was associated with caregiver acceptance of SDF treatment among caregivers born in this country, and lower acculturation was associated with caregiver acceptance of SDF treatment among caregivers born elsewhere.

**Conclusions:**

Caregiver acceptance of SDF treatment is high; child’s age and caregiver comfort are associated with acceptance. Providers need to communicate the risks and benefits of evidence-based dental treatments to increasingly diverse caregiver and patient populations.

## Background

Early childhood caries (ECC) is defined as the presence of 1 or more decayed (non-cavitated or cavitated lesions), missing (due to caries), or filled tooth surfaces in any primary tooth in a child under the age of 6 years [1]. ECC is both multifactorial and highly prevalent among poor and disadvantaged children residing in underprivileged areas due to higher base rates of disease [2]. Moreover, their carious lesions often remain untreated due to limited financial resources and lack of access to dental facilities [3]. Untreated dental caries may result in discomfort, toothache, emergency dental visits, and even hospitalizations; they may also adversely affect weight gain, physical growth, cognitive development, and oral health-related quality of life [4]. Improving the oral health of children is considered a pathway to improving their educational experience, since children with ECC are more likely to miss school and perform poorly due to dental pain [5].

ECC continues to be a social, political, behavioral, and medical problem that can be controlled only through understanding the scope and scale of changes that are taking place in society, particularly those related to the environment such as neighborhood, family structure, nurturing of children, and socioeconomic status [6]. Recent findings based upon data collected as part of the National Health and Nutrition Examination Survey (NHANES) for the years 1999–2004 and 2011–2014 were that caries experience decreased from nearly 42 to 35% and untreated caries decreased from 31 to 18% among preschool-aged children in families with low incomes [7]. Moreover, the proportion of affected carious surfaces may be shifting toward fewer untreated caries to more restored dental surfaces, even as dental caries disparities by poverty status remain for preschool-aged children [8].

Despite documented progress in preventing caries over the past several decades in part due to fluoride application in its various forms [9–12], ECC continues to exact a heavy toll on disadvantaged children, families, and communities. Treatment approaches are shifting away from more invasive surgical drilling of the teeth to remove decay followed by placement of restorations to medical application of preventive chemotherapeutic agents [13]. In particular, interest in aqueous silver diamine fluoride (SDF) has been growing in the United States as both a preventive treatment in community settings and an alternative treatment for caries arrest in the primary dentition and permanent first molars [14]; it was approved for use in Japan over 80 years ago [15, 16]. SDF effectiveness is thought to be due to the combination of silver, which acts as an antimicrobial, fluoride, which promotes remineralization, and ammonia, which stabilizes high concentrations in solution [17].

In 2014, SDF was approved by the US Food and Drug Administration as a treatment for dentinal sensitivity [18]. As recently as 2017, the American Dental Association included a treatment code for interim caries-arresting medicament application (code D1354), and SDF is the sole product on the market [19]. Although SDF treatment of ECC has been shown to be more efficacious when compared with other nonsurgical approaches [20], the side effect of blackening carious lesions (dark marks) is a concern since it is thought to affect caregiver acceptance [21]. Indeed, directors of pediatric residency programs in the United States were surveyed and the most frequently reported barrier to SDF use was parental acceptance because of staining (91.8%) [22].

Results from a randomized clinical trial conducted in Hong Kong using different SDF concentrations and application frequencies indicate that caries arrest treatment by SDF is both effective and safe for preschool-aged children [21, 23, 24]. As expected, staining on arrested carious lesions was common and more so with higher SDF concentration and higher frequency of application, yet parental satisfaction was high and unrelated to the SDF application protocol [21]. The authors argued for studies on parental acceptance of SDF in countries with different cultures to confirm or refute these findings [21].

We posit that improved understanding of the multilevel influences of children’s oral health may lead to the design of more effective and equitable social interventions [25]. For instance, when immigrants move to the United States, they may develop a unique set of cultural norms that blend poor attention to preventive and clinical behaviors from pre-migration heritage with American dietary norms, specifically, often eating highly accessible, cariogenic foods and drinks [26]. In a recent study, maternal oral health behaviors and preferred language were factors significantly associated with ECC in urban Latino children [27]. Less is known about how the sociodemographic and acculturation characteristics of caregivers affect their acceptance of SDF treatment for children with ECC.

### Purpose/objectives

The purpose of this cross-sectional study is to identify factors associated with caregiver acceptance of SDF treatment for children presenting with caries at 7 Federally Qualified Health Centers (FQHCs) in the United States. The objectives are twofold: (1) to examine associations between caregiver acceptance of SDF treatment for children with caries and sociodemographic and acculturation characteristics of caregivers; and (2) to examine associations between caregiver acceptance of SDF treatment for children with caries and clinical assessment of the children by dentists.

## Methods

### Conceptual model

**Fig. 1:**
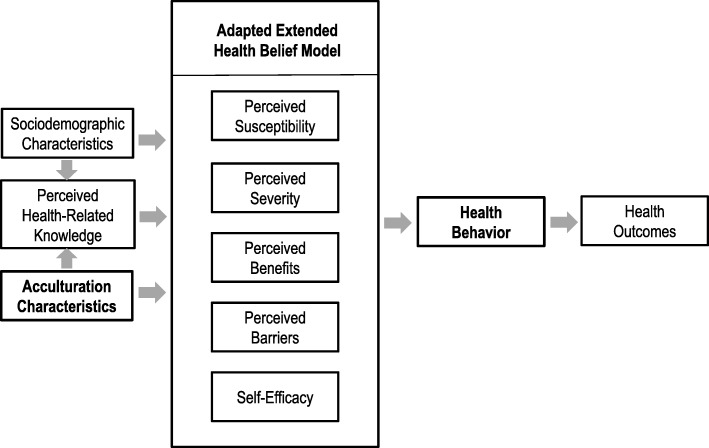
This graphic is an extension of the Health Belief Model [28, 29]. The focus in this schematic as well as in this article is on **acculturation characteristics** that influence the **health behavior** of interest, namely caregiver acceptance of silver diamine fluoride treatment for their children with caries (highlighted through bolding in the figure and legend)

To guide the analyses for this study, we used an extension [28] of the Health Belief Model [29] adapted for the objectives we sought to examine here (Fig. 1).

For the first objective, we view the sociodemographic and acculturation characteristics of caregivers as acting on perceived health-related knowledge, all of which may directly influence perceived susceptibility, perceived severity, perceived benefits, perceived barriers, and self-efficacy to affect health behavior, namely, caregiver acceptance of SDF treatment. The health outcomes of the children are the focus of a related post-treatment study.

For the second objective, we view the clinical assessment of the children by dentists as part of the perceived susceptibility and perceived severity as relayed to the caregivers that affects their health behavior, namely, acceptance of SDF treatment for their children. The Health Belief Model predicts health-related behaviors as a function of beliefs/attitudes only and does not account for other factors known to influence health behaviors, e.g., emotion, cognitive skills, reinforcement (i.e., learning/habit), and environment/context.

### Study design

A cross-sectional survey was conducted in 2017 with caregivers whose children were patients at any of 7 participating community health centers affiliated with the NYU Langone Dental Medicine Pediatric Dentistry Residency Program. Methods employed in this study are consistent with the STROBE guidelines for cross-sectional studies (see www.strobe-statement.org).

### Setting

The 8 community health centers involved in this study are: (1) El Rio Community Health Center, Tucson, AZ (see https://www.aachc.org/community-health-center-locations/member-el-rio-health-center/); (2) Holyoke Health Center, Holyoke, MA (see http://www.hhcinc.org/); (3) Suncoast Community Health Centers, Inc., Brandon, FL (see http://suncoast-chc.org/); (4) Three Lower Counties Community Services, Princess Anne, MD (see http://caroline.md.networkofcare.org/mh/services/agency.aspx?pid=ThreeLowerCountiesCommunityServices_676_2_0); (5) Jordan Valley Community Health Center, Springfield, MO (see https://www.jordanvalley.org/); (6) San Ysidro Health Center, San Diego, CA (see http://www.syhc.org/); (7) Kokua Kalihi Valley Comprehensive Family Services, Honolulu, HI (see http://www.aapcho.org/member/kokua-kalihi-valley-comprehensive-family-services/); and (8) Yakima Valley Farm Worker’s Clinic, Yakima, WA (see http://www.yvfwc.com/). All 8 sites offer a range of medical and dental services to predominantly low-income children, families, and communities, including SDF treatment. A standard protocol for the study was used across sites.

Resident dentists and their faculty mentors at each of the sites received training in the study protocol and collection of data from the Principal Investigator (originally D.C., currently D.M.O.) and conducted all study procedures, including consenting and interviewing the participants, communicating with caregivers regarding SDF treatment for their children, and extracting the data from patient charts / electronic health records (EHRs).

### Participants and study size

All caregivers of pediatric patients who presented with ECC during a routine examination and were diagnosed with 1 or more carious lesions eligible for SDF treatment were informed about the study. Caregivers were eligible to participate in the study if their children were pediatric dental patients at a participating community health center and presented at the center for a pediatric dental visit. Caregiver participants also needed to be able to communicate in English or Spanish. Caregivers who were unable to provide consent or unable to communicate in English or Spanish were ineligible to participate in the study. Verbal consent was obtained from each caregiver prior to completing a survey about caregiver perceptions of SDF treatment in either English or Spanish, according to the stated preference of the participant. Each participating site has interpreter services available in Spanish to aid in translation and ensure understanding of voluntary participation. The Institutional Review Board (IRB) at NYU School of Medicine reviewed and approved all study procedures (s17–00288). IRB approval was obtained in May 2017, and recruitment was ongoing throughout the study. Data collection at the 8 sites ended in November 2017.

Caregiver participants were recruited as follows. Potential caregiver participants bringing their children in for a regularly scheduled dental visit at a participating community health center were introduced to the study by the treating dental resident investigator at the time of their children’s appointments. If the pediatric patient was diagnosed with ECC during the routine exam and it was determined that 1 or more caries were eligible for SDF treatment, the caregiver was provided with further information regarding the study. If the caregiver was interested in participating in the study, verbal consent was administered and a paper survey was provided. The caregiver participants were instructed to deposit their completed surveys in a secure collection box in the waiting area. Each participating community health center had interpreter services available in Spanish, who aided in any needed translation and ensured understanding of voluntary participation. The survey was available in both English and Spanish (translated and back-translated by an outside professional organization). Overall, 546 caregivers across the 8 sites completed the survey and were included in the study.

### Data sources, measurement, and quantitative variables

Data sources included a self-administered survey of caregivers of children with caries and chart reviews to ascertain the type of medical / dental insurance of the pediatric patients, clinical assessments of the pediatric patients by dentists, and whether or not caregivers accepted SDF treatment for their children (see Additional file 1).

#### Sociodemographic characteristics

Survey data were collected on the gender and age of the caregivers, as well as the relationship of the caregivers to the pediatric patients. In addition, caregivers were asked about their Hispanic ethnicity and race from a list of choices, and the highest level of education that they had completed. The insurance (if any) of the pediatric patients was obtained from the chart review.

#### Clinical assessments of pediatric patients by dentists

All pediatric patients in this study received an oral examination, the results of which were recorded in the patients’ charts / EHRs. As a measure of cumulative caries experience, the decayed, missing, filled teeth total scores for primary / permanent teeth (dmft / DMFT) of the pediatric patients were obtained from the chart review. Chart review data were also obtained on whether or not operating room treatment was needed.

#### Caregiver health behavior of interest

The outcome of interest in this study is whether or not the caregivers accepted SDF treatment on the day of survey completion for their children with caries, recorded by the attending dentists in the patients’ charts / EHRs.

#### Acculturation characteristics of caregivers

The Short Acculturation Scale for Hispanics (SASH) score as assessed via the survey was the primary covariate used to measure the acculturation of the caregivers, i.e., the modifications in values, norms, attitudes, and behaviors that occur when immigrants come in contact with a new group, nation, or culture [30]. The SASH has been validated in diverse populations, including in a large sample of breast cancer patients, with oversampling of Latinas and African Americans [31], as well as in Korean immigrants [32]. Individual components of the SASH were also examined, including language caregiver usually speaks at home, language caregiver usually thinks in, and language caregiver usually speaks with friends, where survey responses were: Only English / English better than your native language / Both equally / Your native language better than English / Only your native language. Finally, caregivers were asked if they were born in the United States, and for those who were not born in the United States, how many years they had lived in the United States.

#### Perceived benefits and barriers of caregivers

Likert scales developed specifically for this study were used to measure how likely caregivers were to choose SDF treatment (1 = very unlikely, 5 = very likely), caregiver concern about SDF treatment leaving a permanent dark mark on the tooth (1 = extremely concerned, 5 = not concerned), and caregiver comfort with child receiving SDF treatment (1 = very uncomfortable, 5 = very comfortable).

#### Perceived health-related knowledge of caregivers

A Likert scale developed specifically for this study was also used to measure how much caregivers felt they understood about the SDF treatment that was offered to their children (1 = I do not know anything about it, 5 = I know a lot about it).

### Statistical methods

For descriptive statistics, continuous variables were summarized with means and standard deviations and categorical variables were summarized with counts and percentages, both overall and by age group of pediatric patients (< 6 years, as per the definition of ECC, and > 6 years). Differences by age group of pediatric patients were tested using the non-parametric Wilcoxon rank-sum test for continuous variables (which is robust to outliers and non-normal distributions) and the Fisher exact test for categorical variables, with missing values excluded. Caregiver nativity (US born or not) was initially considered as a potential effect modifier, since the characteristics, benefits and barriers, and knowledge of caregivers may have differential impacts on their acceptance of SDF, which were examined by the significance of two-way interactions in the adjusted models using the likelihood ratio test. A list of the tested interactions along with the results of the statistical tests for these interactions are provided in Additional file 2.

Observations with any missing values in the outcome variable or the covariates of interest were removed before performing any multivariable regression analyses (51 observations or 9% of the total number of 546 observations), leaving a final analytic dataset of *n* = 495. Data from one of the sites on caregiver gender, age, education, SASH score, and understanding of SDF treatment were completely missing (*n* = 31), and hence data from this site represented the majority of observations that were not available for the multivariable analyses.

Standard logistic regression models were developed for caregiver acceptance of SDF treatment for their children as the binary outcome of interest (yes / no) to calculate unadjusted odds ratios (OR) for the potential covariates of interest. The likelihood ratio method was used for hypothesis testing for bivariable associations. Generalized linear mixed models (GLMMs) were developed for multivariable models with caregiver acceptance of SDF treatment for their children as the binary outcome of interest (yes / no) to calculate adjusted ORs. Heterogeneity among the 7 included community health centers was modeled as a random effect in the GLMMs to account for the clustering of observations within sites, enabling inferences to extend to the population of sites. The initial set of variables was selected based upon both theoretical and statistical considerations. Backward variable selection and model comparison were conducted using the likelihood ratio method. Gender, age, education, place of birth (born in the United States or not), and SASH score of the caregiver were considered as confounders and retained in the models whether or not they reached statistical significance. Testing of the random effect was conducted using the parametric bootstrap method. An interaction term (caregiver born in the United States × SASH score) was included in the final GLMM, since it reached statistical significance and explained the complexity of the data. To assess whether or not missing data biased the associations found with caregiver acceptance of SDF treatment, a multiple imputation analysis was conducted, where 20 imputed datasets were created, the multivariable mixed-effect model was fit on each of the imputed datasets, and the results were pooled [33]. All analyses were conducted using the statistical software R version 3.5.1 (https://www.r-project.org/).

## Results

### Participants

The sociodemographic characteristics of the study participants are presented in Table 1.

**Table 1:** Sociodemographic characteristics, clinical assessments, and caregiver behavior of interest [acceptance of silver diamine fluoride (SDF) treatment for pediatric patients]

Characteristics, Assessments, and Behavior	Overall (*n* = 546)	Patients < 6 years (*n* = 410)	Patients ≥6 years (*n* = 136)	*P* Value
Sociodemographic characteristics				
Gender of caregiver				0.628
Male	117 (21.4)	90 (22.0)	27 (19.9)	
Female	397 (72.7)	295 (72.0)	102 (75.0)	
Missing	32 (5.9)	25 (6.1)	7 (5.1)	
Age of caregiver (in years) (*n* = 508)	33.5 ± 9.0	32.5 ± 8.7	36.6 ± 9.0	< 0.001
Relationship of caregiver to pediatric patient				0.100
Parent	509 (93.2)	384 (93.7)	125 (91.9)	
Legal guardian	22 (4.0)	18 (4.4)	4 (2.9)	
Relative (not guardian)	13 (2.4)	6 (1.5)	7 (5.1)	
Other caretaker	2 (0.4)	2 (0.5)	0 (0.0)	
Caregiver of Hispanic or Latino/a ethnicity?				0.053
No	279 (51.1)	197 (48.0)	82 (60.3)	
Yes, Mexican, Mexican American, Chicano/a	160 (29.3)	129 (31.5)	31 (22.8)	
Yes, Puerto Rican	35 (6.4)	25 (6.1)	10 (7.4)	
Yes, Cuban, other Hispanic, Latino/a origin	58 (10.6)	48 (11.7)	10 (7.4)	
Missing	14 (2.6)	11 (2.7)	3 (2.2)	
Race of caregiver				0.045
White	231 (42.3)	180 (43.9)	51 (37.5)	
Black or African American	64 (11.7)	53 (12.9)	11 (8.1)	
American Indian or Alaska Native	11 (2.0)	11 (2.7)	0 (0.0)	
Asian Indian	4 (0.7)	2 (0.5)	2 (1.5)	
Chinese	5 (0.9)	3 (0.7)	2 (1.5)	
Filipino	47 (8.6)	26 (6.3)	21 (15.4)	
Japanese	3 (0.5)	2 (0.5)	1 (0.7)	
Other Asian	9 (1.6)	8 (2.0)	1 (0.7)	
Native Hawaiian or other Pacific Islander	25 (4.6)	14 (3.4)	11 (8.1)	
Chuukese	23 (4.2)	17 (4.1)	6 (4.4)	
Missing	124 (22.7)	94 (22.9)	30 (22.1)	
Education of caregiver				0.352
Less than high school	71 (13.0)	57 (13.9)	14 (10.3)	
High school graduate	230 (42.1)	174 (42.4)	56 (41.2)	
Some college or more	209 (38.3)	150 (36.6)	59 (43.4)	
Missing	36 (6.6)	29 (7.1)	7 (5.1)	
Pediatric patient’s insurance				0.459
Private	20 (3.7)	14 (3.4)	6 (4.4)	
Medicaid (CHIP)	512 (93.8)	387 (94.4)	125 (91.9)	
No insurance	8 (1.5)	6 (1.5)	2 (1.5)	
Other	6 (1.1)	3 (0.7)	3 (2.2)	
Clinical assessments of pediatric patients by dentists				
Decayed, missing, filled teeth total score (dmft / DMFT) (*n* = 512)	7.7 ± 6.2	7.7 ± 6.5	7.8 ± 5.5	0.116
Operating room (OR) treatment needed				< 0.001
Yes	122 (22.3)	107 (26.1)	15 (11.0)	
No	422 (77.3)	301 (73.4)	121 (89.0)	
Missing	2 (0.4)	2 (0.5)	0 (0.0)	
Caregiver health behavior of interest				
Caregiver acceptance of SDF treatment for pediatric patient				< 0.001
Yes	434 (79.5)	344 (83.9)	90 (66.2)	
No	112 (20.5)	66 (16.1)	46 (33.8)	

Continuous variables are presented as mean ± standard deviation. Categorical variables are presented as n (%)

*CHIP* Children’s Health Insurance Program

*P* Values correspond to the testing of differences by age group of pediatric patients using the non-parametric Wilcox rank-sum test for continuous variables (which is robust to outliers and non-normal distributions) and the Fisher exact test for categorical variables, with missing values excluded

The overwhelming majority (93.2%) of the caregiver participants were parents of the pediatric patients with caries and female (72.7%); nearly half (46.3%) were Hispanic and slightly more than half (55.1%) had earned a high school education or less. Nearly all (93.8%) of the pediatric patients with caries were covered by public insurance [Medicaid or Children’s Health Insurance Program (CHIP)], which provide dental benefits up through age 21 years.

### Descriptive data

The acculturation characteristics, perceived benefits and barriers, and perceived health-related knowledge of caregivers regarding SDF treatment for their children with caries are presented in Table 2.

**Table 2:** Acculturation characteristics, benefits and barriers, and health-related knowledge of caregivers, overall and by pediatric patient age group

Characteristics, Benefits and Barriers, and Knowledge of Caregivers	Overall (*n* = 546)	Patients < 6 years (*n* = 410)	Patients ≥6 years (*n* = 136)	*P* Value
Acculturation characteristics of caregivers				
Language caregiver usually speaks at home				0.095
Only English / English more than native	255 (46.7)	186 (45.4)	69 (50.7)	
Both equally	129 (23.6)	92 (22.4)	37 (27.2)	
Native more than English / only native	131 (24.0)	107 (26.1)	24 (17.6)	
Missing	31 (5.7)	25 (6.1)	6 (4.4)	
Language caregiver usually thinks in				0.389
Only English / English more than native	281 (51.5)	205 (50.0)	76 (55.9)	
Both equally	112 (20.5)	83 (20.2)	29 (21.3)	
Native more than English / only native	122 (22.3)	97 (23.7)	25 (18.4)	
Missing	31 (5.7)	25 (6.1)	6 (4.4)	
Language caregiver usually speaks with friends				0.004
Only English / English more than native	270 (49.5)	195 (47.6)	75 (55.1)	
Both equally	116 (21.2)	80 (19.5)	36 (26.5)	
Native more than English / only native	129 (23.6)	110 (26.8)	19 (14.0)	
Missing	31 (5.7)	25 (6.1)	6 (4.4)	
Caregiver born in the United States				0.359
Yes	288 (52.7)	220 (53.7)	68 (50.0)	
No	227 (41.6)	165 (40.2)	62 (45.6)	
Missing	31 (5.7)	25 (6.1)	6 (4.4)	
Years caregiver lived in the United States among those not born in the United States (*n* = 219)	14.4 ± 9.5	13.6 ± 8.8	16.4 ± 11.0	0.107
Short Acculturation Scale for Hispanics (SASH) score (*n* = 515)	3.6 ± 1.5	3.5 ± 1.5	3.8 ± 1.3	0.150
Perceived benefits and barriers of caregivers				
Likelihood of choosing SDF treatment				0.001
Very / somewhat unlikely	95 (17.4)	66 (16.1)	29 (21.3)	
Not sure	94 (17.2)	59 (14.4)	35 (25.7)	
Somewhat / very likely	325 (59.5)	260 (63.4)	65 (47.8)	
Missing	32 (5.9)	25 (6.1)	7 (5.1)	
Concern regarding dark mark of SDF treatment				0.359
Extremely / very concerned	129 (23.6)	92 (22.4)	37 (27.2)	
Moderately / slightly concerned	256 (46.9)	191 (46.6)	65 (47.8)	
Not concerned	159 (29.1)	125 (30.5)	34 (25.0)	
Missing	2 (0.4)	2 (0.5)	0 (0.0)	
Comfort regarding SDF treatment				0.049
Very / somewhat uncomfortable	99 (18.1)	69 (16.8)	30 (22.1)	
Neutral	175 (32.1)	125 (30.5)	50 (36.8)	
Somewhat / very comfortable	271 (49.6)	216 (52.7)	55 (40.4)	
Missing	1 (0.2)	0	1 (0.7)	
Perceived health-related knowledge of caregivers				
Understanding of SDF treatment				0.014
Knows nothing / not enough / not sure	161 (29.5)	107 (26.1)	54 (39.7)	
Knows something	219 (40.1)	170 (41.5)	49 (36.0)	
Knows a lot	135 (24.7)	108 (26.3)	27 (19.9)	
Missing	31 (5.7)	25 (6.1)	6 (4.4)	

Continuous variables are presented as mean ± standard deviation. Categorical variables are presented as n (%)

P Values correspond to the testing of differences by age group of pediatric patients using the non-parametric Wilcox rank-sum test for continuous variables (which is robust to outliers and non-normal distributions) and the Fisher exact test for categorical variables, with missing values excluded

Just over half of the caregivers (52.7%) were born in the United States, and overall the average SASH (acculturation) score was 3.6, where 1 = least acculturated and 5 = most acculturated. Most (59.5%) of the caregivers reported being somewhat or very likely to choose SDF treatment for their children, less than one-third (29.1%) were not concerned about the dark mark, and about half (49.6%) were somewhat or very comfortable with SDF treatment. Understanding of SDF treatment was low among caregiver participants in this study, with only one-quarter (24.7%) reporting that they knew a lot about it.

### Outcome data

Overall, caregiver acceptance of SDF treatment for their children was high (79.5%) (see Table 1). The pediatric patients with caries who presented at community health centers for dental care had substantial caries experience (the average dmft / DMFT score = 7.7) and their dentists believed that 22.3% of them needed OR treatment (see Table 1).

### Main results

A U-shaped relationship between caregiver odds of accepting SDF treatment and age group of pediatric patients was present in the data, where caregivers were most likely to accept SDF treatment for their children who were either < 6 years or 9–14 years, and least likely to accept SDF treatment for pediatric patients 6 to < 9 years (see Table 3).

**Table 3:** Covariates of interest and unadjusted associations of caregiver acceptance of silver diamine fluoride treatment (*n* = 495)

Covariates of Interest	OR	95% CI		*P* Value
Sociodemographic characteristics				
Age group of pediatric patient				
< 6 years	–	–	–	–
6 to < 9 years	0.31	0.18	0.53	< 0.001
9+ years	0.63	0.31	1.37	0.218
Gender of caregiver				
Male	–	–	–	–
Female	0.60	0.33	1.04	0.077
Age of caregiver (in years), continuous	1.02	0.99	1.04	0.236
Education of caregiver				
Less than high school	–	–	–	–
High school graduate	0.43	0.19	0.89	0.031
Some college or more	0.60	0.26	1.26	0.199
Ethnicity of caregiver				
Non-Hispanic	–	–	–	–
Mexican, Mexican American, Chicano/a	2.43	1.39	4.45	0.003
Puerto Rican	0.40	0.19	0.84	0.014
Cuban, other Hispanic, Latino/a origin	1.01	0.48	2.27	0.980
Clinical assessments of child patients by dentists				
Decayed, missing, filled teeth total score (dmft or DMFT), continuous	1.06	1.02	1.12	0.007
Operating room (OR) treatment needed				
No	–	–	–	–
Yes	2.24	1.26	4.28	0.009
Acculturation characteristics of caregivers				
Caregiver born in the United States				
No	–	–	–	–
Yes	1.26	0.82	1.94	0.284
Short Acculturation Scale for Hispanics (SASH) score, continuous	0.91	0.78	1.06	0.236
Perceived benefits and barriers of caregivers				
Likelihood of choosing silver diamine fluoride (SDF) treatment				
Very / somewhat unlikely	–	–	–	–
Not sure	0.96	0.53	1.74	0.884
Somewhat / very likely	4.88	2.84	8.42	< 0.001
Concern regarding dark mark of SDF treatment				
Not concerned	–	–	–	–
Moderately / slightly concerned	0.40	0.20	0.77	0.009
Extremely / very concerned	0.14	0.07	0.27	< 0.001
Comfort regarding SDF treatment				
Very / somewhat uncomfortable	–	–	–	–
Neutral	2.44	1.41	4.26	0.001
Somewhat / very comfortable	11.8	6.40	22.5	< 0.001
Perceived health-related knowledge of caregivers				
Understanding of SDF treatment				
Knows nothing / not enough / not sure	–	–	–	–
Knows something	2.96	1.80	4.92	< 0.001
Knows a lot	2.91	1.66	5.23	< 0.001

For categorical variables, ORs are presented where the 1st level is the reference category

For continuous variables (labeled as such), ORs are presented for a 1-unit increase

*OR* odds ratio, *CI* confidence interval

Caregivers were also more likely to accept SDF treatment for their children with substantial caries experience, i.e., high dmft / DMFT scores, and if their dentists believed that OR treatment was needed.

### Multivariable analyses

The final adjusted multivariable model for caregiver acceptance of SDF treatment for their children with caries is presented in Table 4.

**Table 4:** Covariates of interest and adjusted associations of caregiver acceptance of silver diamine fluoride treatment. (*n* = 495)

Covariates of Interest	Adjusted OR	95% CI		*P* Value
Age group of pediatric patient				
< 6 years	–	–	–	–
6 to < 9 years	0.38	0.17	0.82	0.014
9+ years	1.86	0.68	5.08	0.226
Gender of caregiver				
Male	–	–	–	–
Female	0.75	0.36	1.54	0.430
Age of caregiver (in years), continuous	1.02	0.99	1.06	0.212
Education of caregiver				
Less than high school	–	–	–	–
High school graduate	0.74	0.27	2.02	0.563
Some college or more	1.06	0.37	3.02	0.917
Decayed, missing, filled teeth total score (dmft or DMFT), continuous	1.08	1.00	1.16	0.038
Caregiver born in the United States				
No	–	–	–	–
Yes	0.19	0.02	1.81	0.150
Short Acculturation Scale for Hispanics (SASH) score, continuous	0.67	0.44	1.03	0.071
Caregiver born in the United States X SASH score (interaction term)	1.84	1.02	3.32	0.042
Likelihood of choosing SDF treatment				
Very / somewhat unlikely	–	–	–	–
Not sure	1.58	0.69	3.63	0.280
Somewhat / very likely	3.07	1.50	6.28	0.002
Concern regarding dark mark of SDF treatment				
Not concerned	–	–	–	–
Moderately / slightly concerned	0.59	0.26	1.33	0.204
Extremely / very concerned	0.33	0.14	0.80	0.014
Comfort regarding SDF treatment				
Very / somewhat uncomfortable	–	–	–	–
Neutral	2.34	1.13	4.86	0.022
Somewhat / very comfortable	6.19	2.74	14.0	< 0.001
Understanding of SDF treatment				
Knows nothing / not enough / not sure	–	–	–	–
Knows something	2.59	1.33	5.05	0.005
Knows a lot	1.86	0.83	4.15	0.130

Adjusted OR = odds ratio adjusted for the other predictors in the model

*CI* confidence interval

For categorical variables, ORs are presented where the 1st level is the reference category

For continuous variables (labeled as such), ORs are presented for a 1-unit increase

Adjusting for the effects of the other factors in the model, among caregivers born in the United States, a 1-unit increase in SASH score increased the odds of caregiver acceptance of SDF treatment by a factor of 1.2. On the other hand, among caregivers not born in the United States, a 1-unit increase in SASH score decreased the odds of caregiver acceptance of SDF treatment by a factor of 0.7. This result is largely driven by the subgroup of caregiver participants with a SASH score = 1 (least acculturated). Only 5 caregivers with a SASH score = 1 were born in the United States; of these, 3 accepted SDF treatment (60.0%). Yet of the 46 caregivers with a SASH score = 1 born outside of the United States, 45 accepted SDF treatment (97.8%).

The strongest association in the final model for caregiver acceptance of SDF treatment for their children was with caregiver comfort with SDF treatment. Adjusted for the effects of the other factors in the model, the odds of caregiver acceptance of SDF treatment among those who were neutral regarding SDF treatment was 2.3 times that for those who were very or somewhat uncomfortable with SDF treatment; the odds of caregiver acceptance of SDF treatment among those who were somewhat or very comfortable regarding SDF treatment was 6.2 times that for those who were very or somewhat uncomfortable.

For the 3 sociodemographic characteristics of the caregivers in the final model (gender, age, and education), all of the associated 95% confidence intervals (CIs) around the adjusted ORs included 1. Hispanic ethnicity was not included in the final model for 2 reasons. First, the goodness of fit was improved without Hispanic ethnicity in the model. Second, SASH score was moderately correlated with Hispanic ethnicity, but SASH score was more strongly associated with caregiver acceptance of SDF treatment, especially when the interaction term between caregiver born in the United States and SASH score was included in the model. The parametric bootstrap test for the random effect had a *p*-value < 0.001, indicating significant variability among the 7 sites in caregiver acceptance.

Although the likelihood of choosing SDF treatment was correlated with caregiver acceptance of SDF treatment, important differences were found in participant responses and behaviors as measured by these two variables. Indeed, of the 93 caregivers who initially reported that it was very or somewhat unlikely that they would choose SDF treatment, 57 (61%) actually accepted SDF treatment for their children. Results using the pooled results from multiple imputation [33] with 20 datasets (Table 5) confirmed the original multivariable results (Table 4), meaning that missing values did not bias the substantive findings obtained, which are robust to the statistical approaches employed.

**Table 5:** Covariates of interest and adjusted associations of caregiver acceptance of silver diamine fluoride treatment using multivariate imputation by chained equations (MICE) (*n* = 495)

Covariates of Interest	Adjusted OR	95% CI		*P* Value
Age group of pediatric patient				
< 6 years	–	–	–	–
6 to < 9 years	0.34	0.16	0.72	0.002
9+ years	1.82	0.68	4.87	0.365
Gender of caregiver				
Male	–	–	–	–
Female	0.77	0.39	1.55	0.465
Age of caregiver (in years), continuous	1.02	0.98	1.05	0.360
Education of caregiver				
Less than high school	–	–	–	–
High school graduate	0.78	0.29	2.08	0.618
Some college or more	1.08	0.39	3.02	0.882
Decayed, missing, filled teeth total score (dmft or DMFT), continuous	1.08	1.01	1.16	0.025
Caregiver born in the United States				
No	–	–	–	–
Yes	0.10	0.01	0.78	0.028
Short Acculturation Scale for Hispanics (SASH) score, continuous	0.69	0.45	1.05	0.086
Caregiver born in the United States X SASH score (interaction term)	2.07	1.19	3.60	0.010
Likelihood of choosing SDF treatment				
Very / somewhat unlikely	–	–	–	–
Not sure	1.69	0.74	3.88	0.214
Somewhat / very likely	2.89	1.42	5.88	0.003
Concern regarding dark mark of SDF treatment				
Not concerned	–	–	–	–
Moderately / slightly concerned	0.61	0.27	1.37	0.231
Extremely / very concerned	0.32	0.13	0.76	0.010
Comfort regarding SDF treatment				
Very / somewhat uncomfortable	–	–	–	–
Neutral	2.34	1.14	4.78	0.021
Somewhat / very comfortable	6.77	3.02	15.2	< 0.001
Understanding of SDF treatment				
Knows nothing / not enough / not sure	–	–	–	–
Knows something	2.74	1.42	5.31	0.003
Knows a lot	1.89	0.85	4.21	0.120

Adjusted OR = odds ratio adjusted for the other predictors in the model

*CI* confidence interval

For categorical variables, ORs are presented where the 1st level is the reference category

For continuous variables (labeled as such), ORs are presented for a 1-unit increase

## Discussion

### Key results

Among the group of caregivers and their children with caries who presented at community health centers for dental care, caregiver acceptance of SDF treatment was high (79.5%), especially for children with ECC and those with higher caries experience (dmft / DMFT scores). This is understandable, since the current standard of care for treatment of severe ECC usually necessitates general anesthesia with all of its potential complications [34], given the lack of cooperative behavior of infants and preschool-aged children [35].

The findings regarding caregiver acculturation and acceptance of SDF treatment for their children with caries are both novel and complex. Effect modification was present in our data, such that the relationship between acculturation and caregiver acceptance of SDF treatment depended upon whether or not caregivers were born in the United States. If they were, then greater acculturation was associated with caregiver acceptance of SDF treatment, perhaps because more acculturated caregiver participants are concerned about the impact of general anesthesia. If they were born elsewhere, then lower acculturation was associated with caregiver acceptance of SDF treatment. This result is largely driven by the least acculturated participants. The high percentage of caregiver acceptance of SDF treatment among those born outside of the United States with low acculturation may be due to a tendency to accept noninvasive treatments such as SDF application in their countries of origin. Once caregivers born outside of the United States become more acculturated, they may become increasingly attracted to highly technical and invasive dental procedures that are the norm in this country. These potential explanations are speculative only, and may in fact be data driven, requiring additional study and confirmation.

### Interpretation

The findings reported here are consistent with growing support for SDF treatment for caries in primary teeth, including parental satisfaction with the aesthetic outcome and pediatric patient acceptance and comfort [36, 37]. Moreover, recent systematic reviews have found that SDF applications are effective in preventing caries in the primary dentition [38] and in arresting caries in the exposed root surfaces of older adults [39], making SDF a simple, inexpensive, and safe way of preventing caries initiation and progression across the life-course.

Acculturation also plays a key role in health status and promotion, since immigrants who have lived in the United States for longer periods of time and possess higher degrees of acculturation may have different lifestyles than those who report fewer years of US residence or lesser degrees of acculturation [40]. The complex findings in our data were largely driven by the least acculturated caregiver participants and require confirmation in subsequent studies.

The result regarding increased caregiver acceptance of SDF treatment for children 9–14 years compared to children 6 to < 9 years is novel, likely because most studies of caregiver acceptance of SDF treatment have been conducted in younger children. Nonetheless, this finding appears plausible, since primary teeth in children 9–14 years will soon fall out. The U-shaped relationship between caregiver odds of accepting SDF treatment and age group of pediatric patients reported here may be data driven, and needs further testing and confirmation, including whether primary or permanent teeth / anterior or posterior teeth required treatment for carious lesions.

### Limitations

Several limitations of this study deserve comment. First, while the participating dentists used a standard protocol across the 8 sites, data were not collected on the encouragement of caregivers by dental providers to accept SDF treatment for their pediatric patients; data were also not collected on whether risk and severity were always communicated, uniformly and with ensured caregiver understanding. Second, dentists did not record the number of caregivers approached nor any of the characteristics of those who refused to complete the survey that would have permitted comparison between study decliners and completers or assessment of response bias. Third, the dmft / DMFT scores were obtained from the patients’ charts / EHRs, with no effort to determine their accuracy or reliability, given limited resources. Finally, greater caregiver acceptance of SDF treatment for caries on children’s posterior teeth (premolars and molars) than for caries on children’s anterior teeth (incisors and canine teeth) has been reported [41], but this potential covariate (anterior / posterior tooth location of carious lesion) was not initially considered and thus was not explicitly included in the patients’ charts / EHRs.

### Generalizability

Study participants were caregivers of children 14 years and younger who presented for dental care due to caries at community health centers, the vast majority of whom were covered by public insurance (Medicaid / CHIP). Hence, findings may not be generalizable to caregivers and their children with caries who are covered by private insurance or did not seek care for childhood dental caries, including undocumented immigrants who are either ineligible for care or forced to forgo care because they fear interactions with public agencies [42].

## Conclusion

Caregiver acceptance of SDF treatment is high, and child’s age and caregiver comfort are associated with acceptance. Given the complex interplay of acculturation with country of birth, providers need to be prepared to communicate the risks and benefits of evidence-based dental treatments to increasingly diverse caregiver and patient populations.

## Supplementary information

**Additional file 1.** Questionnaire I and Chart review I. A self-administered survey of caregivers of children with caries and a chart review to ascertain the type of medical / dental insurance of the pediatric patients, clinical assessments of the pediatric patients by dentists, and whether or not caregivers accepted silver diamine fluoride treatment for their children.

**Additional file 2.** Statistical appendix. Statistical methods to examine interactions and the proportion of study participants relative to the pediatric patient pools across the involved clinics.

## Data Availability

De-identified raw data and materials described in the manuscript are freely available from the corresponding author on reasonable request.

## Abbreviations

dmft / DMFT: Decayed, missing, filled teeth
ECC: Early childhood caries
EHR: Electronic health records
FQHC: Federally Qualified Health Center
IRB: Institutional Review Board
NHANES: National Health and Nutrition Examination Survey
OR: Odds ratio
SASH: Short Acculturation Scale for Hispanics
SDF: Silver diamine fluoride
