# Supplementary material for: A survey of caregiver acculturation and acceptance of silver diamine fluoride treatment for childhood caries
Source: BMC Oral Health. 2019 Oct 24;19:228. doi: 10.1186/s12903-019-0915-1 (PMC6814040; doi:10.1186/s12903-019-0915-1)
Supplement: Supplementary file 1 — Additional file 1. Questionnaire I and Chart review I. A self-administered survey of caregivers of children with caries and a chart review to ascertain the type of medical / dental insurance of the pediatric patients, clinical assessments of the pediatric patients by dentists, and whether or not caregivers accepted silver diamine fluoride treatment for their children. [file 12903_2019_915_MOESM1_ESM.pdf]

Date: __________ Participant ID: ________
Interviewer initials: ________ Health Center: _______________

<u>Questionnaire I</u>: To be given to parents of children eligible for SDF treatment.

The purpose of this research survey is to evaluate parental perceptions of silver diamine fluoride treatment for dental caries in children. It is being conducted by <u>(fill in name of health center here)</u>, an affiliate of NYU Lutheran Department of Dental Medicine. You are invited to participate in this research project because you are the parent or caregiver to a child aged 0-14 years old coming in for a dental visit today.

Your participation in this research survey is voluntary. You may choose not to participate. If you decide to participate in this research survey, please answer the following questions. If you decide not to participate in this survey, your child's care will not be affected.

Your responses to this survey will be confidential and we do not collect identifying information such as your name, email address or IP address. The survey questions will be about you and your child's demographic information as well as oral health questions.

We will do our best to keep your information confidential. All data is stored in a password protected electronic format. To help protect your confidentiality, the surveys will not contain information that will personally identify you. The results of this study will be used for scholarly purposes only.

If you have any questions about this survey, feel free to speak with <u>(name of resident conducting research</u>). This research has been reviewed according to NYU School of Medicine Institutional Review Board procedures for research involving human subjects.

Completing the following survey indicates that:

- You have read the above information
- You voluntarily agree to participate
- You are at least 18 years of age

If you do not wish to participate in the research study, please do not complete the survey.

Thank you for participating in this study about your child's visit today. This survey is 22 questions and will ask you about yourself and a little about a treatment for your child's dental caries, some questions about yourself and your family.

1) What is **<u>your</u>** age? _______ years

2) What is **<u>your</u>** gender? 1☐Male 2☐Female 3☐ Other

3) What is your relationship to the child being seen today?

1☐Parent 2☐Legal Guardian 3☐ Relative (not guardian) 4☐Other caretaker 5☐Refuse

4) Are you Hispanic or Latino?

1. ☐No
2. ☐Yes, Mexican, Mexican American, Chicano/a
3. ☐Yes, Puerto Rican
4. ☐Yes, Cuban
5. ☐Yes, other Hispanic, Latino/a origin
6. ☐Refuse

5) Which one of these groups would you say best represents your race?

1.☐White	6. ☐Filipino
2.☐Black or African American	7. ☐Japanese
3.☐American Indian or Alaska Native	8. ☐Other Asian
4.☐Asian Indian	9. ☐Native Hawaiian or Other Pacific Islander
5.☐ Chinese	10.☐Don't know/Not sure

6) How many people currently live in your household, including yourself? 1. __ __

7) How many children less than 18 years of age live in your household? 1. __ __

8) What is the highest level of education that you have completed?

1.☐No formal education	6. ☐Some college
2.☐Elementary (primary school) education	7. ☐College graduate
3.☐Middle school (junior high school) education	8. ☐Some graduate school or more
4.☐Some high school	9. ☐Don't know/Not sure
5.☐High school graduate/ equivalency (GED)	10.☐Refuse

Today your dentist spoke with you about a treatment for your child's caries, called silver diamine fluoride or SDF. It is a treatment for the cavities in your child's tooth or teeth. This treatment is used often by dentists on children, but it leaves a permanent dark mark on the tooth where the treatment is placed. The next few questions are about how you feel about treatment with SDF and whether you will agree to this treatment for your child.

9) Do you know of any other child who has had silver diamine fluoride (SDF) treatment (such as a child in your family, friend or relative)?

1☐No 2☐ Yes 3☐Don't know

10) Has your child been treated for caries before?

1☐No 2☐ Yes 3☐Don't know

Date: ___________ Participant ID: _________
Interviewer initials: ________ Health Center: ________________

11) After your child's dentist has described silver diamine fluoride to you, how likely are you to choose this method to treat your child's treatment today?

1. ☐Very unlikely	2. ☐Somewhat unlikely	3. ☐Not sure	4.☐ Somewhat likely	5.☐ Very likely

12) This treatment leaves a permanent dark mark on the tooth, how much is this concern for you?

1. ☐ Extremely concerned	2. ☐Very concerned	3. ☐Moderately concerned	4. ☐Slightly concerned	5. ☐ Not concerned

13) If the dark mark is not a concern, what is the primary reason why?
1. ☐The dark mark will not be visible when my child smiles.
2. ☐The dark mark is temporary until my child is seen for their procedure.
3. ☐The dark mark does not concern me or my child.
4. ☐I don't like the dark mark, but I feel the treatment is the best.

14) How do you feel about your child receiving SDF treatment today?

1. ☐ Very uncomfortable	2. ☐ Somewhat uncomfortable	3. ☐ Neutral	4. ☐ Somewhat comfortable	5. ☐ Very comfortable

15) How much do you feel you understand about the SDF treatment that was offered to your child today?
1. ☐I do not know anything about it
2. ☐I don't know enough about it to fully understand it
3. ☐I'm not sure whether or not I know enough about this
4. ☐I know something about it, but I want to know more about it
5. ☐I know a lot about it

The next few questions ask about your language habits at home.

16) What is your native language?

1. ☐English	2. ☐Spanish
3. ☐Tagalog	4. ☐Ilocano
5. ☐Chukeese	6. ☐Other: ______

Date: ___________ Participant ID: _________
Interviewer initials: ________ Health Center: ________________

17) In general, what language(s) do you read and speak?
    1. ☐Only English
    2. ☐English better than your native language
    3. ☐Both equally
    4. ☐Your native language better than English
    5. ☐Only your native language

18) What language do you usually speak at home?
    1. Only English
    2. English better than your native language
    3. Both equally
    4. Your native language better than English
    5. Only your native language

19) In what language do you usually think?
    1. ☐Only English
    2. ☐English better than your native language
    3. ☐Both equally
    4. ☐Your native language better than English
    5. ☐Only your native language

20) In what language do you usually speak with your friends?
    1. ☐Only English
    2. ☐English better than your native language
    3. ☐Both equally
    4. ☐Your native language better than English
    5. ☐Only your native language

21) Were you born in the United States?

1.☐Yes 2. ☐ No

22) If you were not born in the United States, how many years have you lived here? 1. ______ years

Date: ___________ Participant ID: _________
Interviewer initials: ________ Health Center: ________________

Questionnaire I: Chart review

CR1: Patient's age (months): ________

CR2: Patient's gender: ________

CR3: Patient's insurance:

1. ☐Private
2. ☐Medicaid (CHIP)
3. ☐No insurance
4. ☐Other: ___________________

CR4: ASA level:

1. ☐ I 2. ☐ II 3. ☐ III

CR5: Co-morbidities: 1. _________________ 2. ___________________ 3. _

_____________________ 4._________________ 5.____________________

CR6: DMFT or dmft total score: _____________

CR7: SDF treated caries location(s):

Tooth1: __________<br>Surface1: __________	Tooth2: __________<br>Surface2: __________	Tooth3: __________<br>Surface3: __________	Tooth4: __________<br>Surface4: __________
Tooth5: __________<br>Surface5: __________	Tooth6: __________<br>Surface6: __________	Tooth7: __________<br>Surface7: __________	Tooth8: __________<br>Surface8: __________
Tooth9: __________<br>Surface9: __________	Tooth10: __________<br>Surface10: __________	Tooth11: __________<br>Surface11: __________	Tooth12: __________<br>Surface12: __________

CR8: Is OR treatment needed?

1. ☐Yes 2. ☐ No

CR9: Is sedation needed?

1. ☐Yes 2. ☐ No

CR10: a. When is the OR visit: _______________ or b. Sedation visit? __________________

CR11: Did the parent accept SDF treatment today?

1. ☐Yes 2. ☐ No
