# Supplementary material for: A survey of caregiver acculturation and acceptance of silver diamine fluoride treatment for childhood caries
Source: BMC Oral Health. 2019 Oct 24;19:228. doi: 10.1186/s12903-019-0915-1 (PMC6814040; doi:10.1186/s12903-019-0915-1)
Supplement: Supplementary file 2 — Additional file 2. Statistical appendix. Statistical methods to examine interactions and the proportion of study participants relative to the pediatric patient pools across the involved clinics. [file 12903_2019_915_MOESM2_ESM.docx]

**Additional file 2: Statistical appendix**

*Statistical methods to examine interactions*

The authors initially considered whether or not born in the United States (born_US) could potentially interact with the characteristics, benefits and barriers, and knowledge of caregivers, including the Short Acculturation Scale for Hispanics (SASH), “Concern regarding dark mark of SDF treatment” (dark_mark_concern), “Understanding of SDF treatment” (understanding_SDF), “Comfort regarding SDF treatment” (comfort_with_SDF), and “Likelihood of choosing SDF treatment” (likelihood_of_choosing_SDF). Interactions of born_US and each of these variables were examined in separate generalized linear mixed models (GLMMs).

The table below presents the P Values of the 2-way interactions provided by the likelihood ratio test for interactions between born_US and the five variables listed above (SASH, dark_mark_concern, understanding_SDF, comfort_with_SDF, and likelihood_of_choosing_SDF).

| **Interaction** | **P Value** |
| --- | --- |
| born_US * SASH | 0.045 |
| born_US * dark_mark_concern | 0.850 |
| born_US * understanding_SDF | 0.710 |
| born_US * comfort_with_SDF | 0.966 |
| born_US * likelihood_of_choosing_SDF | 0.224 |

Another way to demonstrate the interaction between the variables born_US and SASH is to present a table of linear contrasts based upon the fitted model. The odds ratios presented below estimate the effect of born_US on caregiver acceptance of SDF treatment at each of the five levels of the SASH score.

| **SASH score** | **Odds Ratio** | **95% Confidence Interval** | | **P Value** |
| --- | --- | --- | --- | --- |
| 1 | 0.4 | 0.1 | 2.0 | 0.234 |
| 2 | 0.7 | 0. 2 | 2.2 | 0.496 |
| 3 | 1.2 | 0.5 | 2.9 | 0.679 |
| 4 | 2.2 | 0.9 | 5.3 | 0.07 |
| 5 | 4.1 | 1.2 | 13.4 | 0.02 |

When the SASH score is less than 4 (representing the least acculturated caregivers), a null effect of born_US on caregiver acceptance of SDF treatment is observed. On the other hand, when the SASH score is 4 or greater (representing the most acculturated caregivers), there is evidence of an effect of born_US on caregiver acceptance of SDF treatment, such that the odds of accepting SDF for caregivers born in the United States is 2.2 [0.9, 5.3] times higher than that for those born outside of the United States if the SASH score = 4, and 4.1 [1.2, 13.4] times higher if the SASH score = 5.

A final way to present the interaction between the variables born_US and SASH is visually through the figure presented on the following page. For caregivers born in the United States, the odds of accepting SDF treatment in increased with higher SASH scores, but this relationship is reversed for caregivers born outside of the United States.


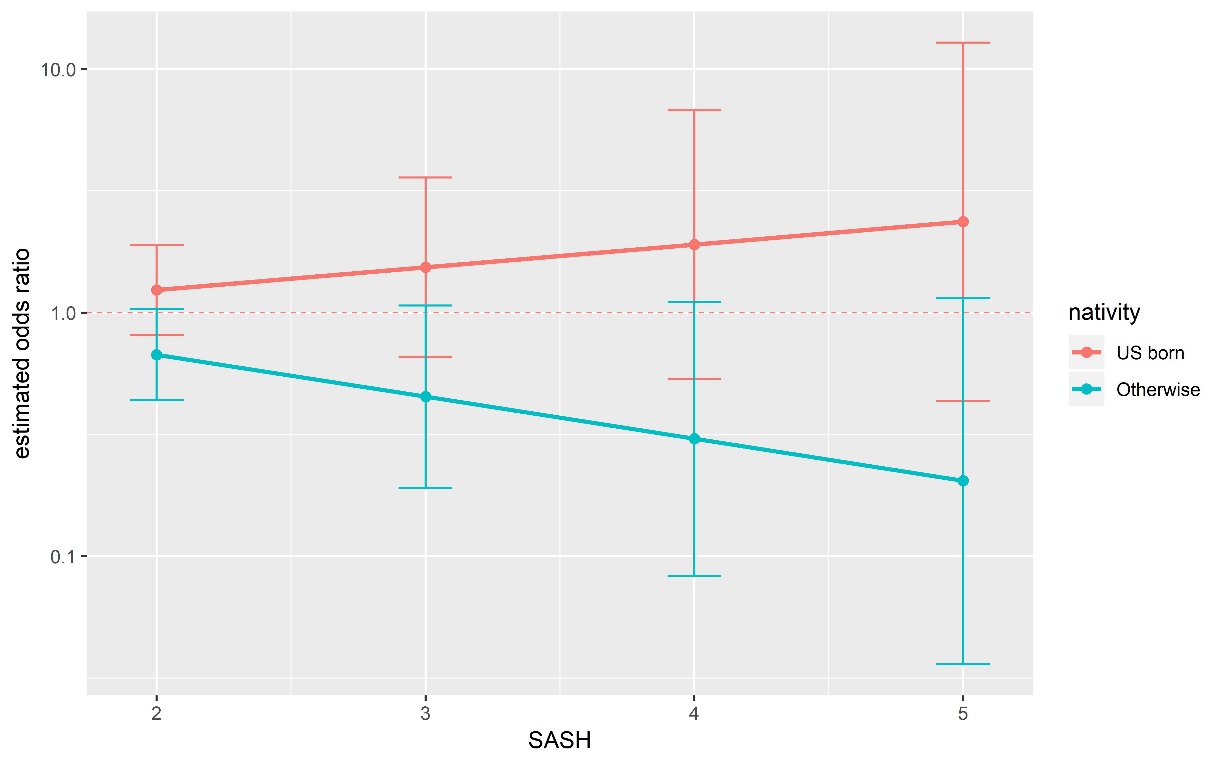


**Figure.** Estimated odds ratio of caregiver acceptance of SDF treatment for higher SASH scores versus the lowest SASH score (SASH=1) from the final adjusted generalized linear mixed model. Note that the Y-axis representing the estimated odds ratio is presented on a log 10 scale for improved visualization. The X-axis represents the SASH score. Each solid point with a vertical error bar represents an estimated odds ratio and 95% confidence interval, respectively.

*Proportion of study participants relative to the pediatric patient pools across clinics*

Using the Health Resources and Services Administration (HRSA) Health Center Program site and accessing Health Center Data (see https://bphc.hrsa.gov/datareporting/index.html), we were able to locate 6 of the 8 participating community health centers with data available for 2017 (the year the study was conducted). Using the available data for the number of children (< 18 years old) for Year 2017 by clinic, and the number of study participants per clinic, we were able to compute the proportion of study participants relative to the pediatric patient pools across clinics (see the table below).

| **Community health center** | **El Rio Community Health Center** | **Holyoke Health Center** | **Suncoast Community Health Centers** | **Three Lower Counties Community Services** | **Kokua Kalihi Valley Comprehensive Family Services** | **Yakima Valley Farm Worker’s Clinic** |
| --- | --- | --- | --- | --- | --- | --- |
| **Pediatric patient pool** | 34,438 | 6,246 | 26,370 | 14,217 | 4,587 | 61,332 |
| **Study participants** | 61 | 34 | 127 | 36 | 95 | 31 |
| **Study participants / Pediatric patient pool** | 0.02% | 0.05% | 0.05% | 0.03% | 2.1% | 0.05% |

As shown in the table above, the proportion of study participants / pediatric patient pool ranged from 0.02% to 2.1% across the 6 clinics.
